# Myc interacts with Max and Miz1 to repress C/EBPδ promoter activity and gene expression

**DOI:** 10.1186/1476-4598-9-92

**Published:** 2010-04-28

**Authors:** Junling Si, Xueyan Yu, Yingjie Zhang, James W DeWille

**Affiliations:** 1 Department of Veterinary Biosciences, Ohio State University College of Veterinary Medicine, Columbus Ohio, 43210, USA; 2 OSU Comprehensive Cancer Center, 1925 Coffey Road, Columbus Ohio, 43210, USA

## Abstract

**Background:**

"Loss of function" alterations in CCAAT/Enhancer Binding Proteinδ (C/EBPδ) have been reported in a number of human cancers including breast, prostate and cervical cancer, hepatocellular carcinoma and acute myeloid leukemia. C/EBPδ gene transcription is induced during cellular quiescence and repressed during active cell cycle progression. C/EBPδ exhibits tumor suppressor gene properties including reduced expression in cancer cell lines and tumors and promoter methylation silencing.

We previously reported that C/EBPδ expression is inversely correlated with c-Myc (Myc) expression. Aberrant Myc expression is common in cancer and transcriptional repression is a major mechanism of Myc oncogenesis. A number of tumor suppressor genes are targets of Myc transcriptional repression including C/EBPα, p15^*INK*4^, p21^*CIP*1^, p27^*KIP*1 ^and p57^*KIP*2^. This study investigated the mechanisms underlying Myc repression of C/EBPδ expression.

**Results:**

Myc represses C/EBPδ promoter activity in nontransformed mammary epithelial cells in a dose-dependent manner that requires Myc Box II, Basic Region and HLH/LZ domains. Chromatin Immunoprecipitation (ChIP) assays demonstrate that Myc, Miz1 and Max are associated with the C/EBPδ promoter in proliferating cells, when C/EBPδ expression is repressed. EMSAs demonstrate that Miz1 binds to a 30 bp region (-100 to -70) of the C/EBPδ promoter which contains a putative transcription initiator (Inr) element. Miz1 functions exclusively as a repressor of C/EBPδ promoter activity. Miz1 siRNA expression or expression of a Miz1 binding deficient Myc (MycV394D) construct reduces Myc repression of C/EBPδ promoter activity. Max siRNA expression, or expression of a Myc construct lacking the HLH/LZ (Max interacting) region, also reduces Myc repression of C/EBPδ promoter activity. Miz1 and Max siRNA treatments attenuate Myc repression of endogenous C/EBPδ expression. Myc Box II interacting proteins RuvBl1 (Pontin, TIP49) and RuvBl2 (Reptin, TIP48) enhances Myc repression of C/EBPδ promoter activity.

**Conclusion:**

Myc represses C/EBPδ expression by associating with the C/EBPδ proximal promoter as a transient component of a repressive complex that includes Max and Miz1. RuvBl1 and RuvBl2 enhance Myc repression of C/EBPδ promoter activity. These results identify protein interactions that mediate Myc repression of C/EBPδ, and possibly other tumor suppressor genes, and suggest new therapeutic targets to block Myc transcriptional repression and oncogenic function.

## Background

CCAAT/Enhancer Binding Proteinδ (C/EBPδ) is a member of the highly conserved C/EBP family of leucine zipper DNA binding proteins [1-3]. C/EBPδ gene expression is increased in nontransformed mammary epithelial cells (MECs) in response to G_0 _growth arrest conditions and IL-6 family cytokine treatment [4-11]. Ectopic C/EBPδ expression induces growth arrest of mammary epithelial, prostate and chronic myelogenous leukemia cell lines [5,12,13]. Conversely, reducing C/EBPδ gene expression is associated with delayed growth arrest, genomic instability, impaired contact inhibition, increased cell migration and increased growth in reduced serum media [5,14]. *In vivo*, female C/EBPδ knockout mice exhibit increased mammary epithelial cell proliferation and mammary gland ductal hyperplasia [15].

"Loss of function" alterations in C/EBPδ gene expression have been reported in human and experimental cancer. Using Serial Analysis of Gene Expression (SAGE) assays Polyak and coworkers demonstrated that C/EBPδ is down regulated in the progression from normal breast epithelium to advanced breast cancer [16,17]. Other reports have shown that C/EBPδ gene expression is reduced in ~30% of primary human breast tumors and in primary prostate tumors [11,18]. In experimental models, C/EBPδ expression is reduced in carcinogen-induced mammary tumors and in ~50% of mammary tumors isolated from MMTV/c-neu transgenic mice [19,20].

Studies addressing the mechanisms underlying loss of function alterations in C/EBP gene expression demonstrated that the C/EBPδ gene promoter is silenced by promoter hypermethylation in the SUM-52PE human breast cancer cell line (26/27 CpGs methylated) and by site-specific methylation in primary human breast tumor isolates [11]. C/EBPδ gene expression is also silenced by promoter hypermethylation in primary cervical cancer and hepatocellular carcinoma (HCC) [21]. In addition to solid tumors, C/EBPδ gene expression is reduced and the C/EBPδ promoter is silenced by hypermethylation in the U937 human lymphoma derived cell line and in ~35% of lymphoma cells isolated from AML patients [22]. Although C/EBPδ expression is reduced in primary tumors and cancer derived cell lines inactivating mutations in the intronless C/EBPδ gene are rare [23,24]. This indicates that alterations in regulatory mechanisms that control C/EBPδ gene expression play a key role in cancer-related C/EBPδ "loss of function" alterations. We used nuclear run-on assays to investigate C/EBPδ transcriptional regulation and found that C/EBPδ gene transcription is induced ~6 fold in G_0 _growth arrested nontransformed mammary epithelial cells compared to actively proliferating mammary epithelial cells [6]. These findings demonstrated the importance of transcriptional control of C/EBPδ gene expression and suggested that alterations in transcriptional activators or repressors would have a major impact on C/EBPδ expression and cellular growth control.

c-Myc (Myc) is a member of the Myc family of helix loop helix proteins that function in the activation and repression of target gene transcription [25]. Myc expression promotes cell proliferation and Myc over expression has been documented in a wide range of human cancers [25]. The Myc gene is frequently amplified in breast cancer and experimental studies indicate that Myc is a downstream transcriptional effector of ErbB2 receptor tyrosine kinase activation, a signaling pathway that is commonly dysregulated and constitutively active in breast cancer [26,27]. Accumulating evidence indicates that transcriptional repression of Myc target genes is a major mechanism in which Myc promotes cell transformation [28]. Myc represses the transcription of key growth control, differentiation and tumor suppressor genes including GAS1, p15^*INK*4^, p21^*CIP*1^, p27^*KIP*1^, p57^*KIP*2^, growth arrest and DNA damage 34 (GADD34), GADD45, C/EBPα and GADD153 (C/EBPζ) [25,28-42].

We previously reported that the C/EBPδ proximal promoter is in a constitutively "open" chromatin conformation and that the C/EBPδ proximal promoter is accessible to activating (Sp1, pSTAT3, CREB) and repressive (Myc) transcriptional regulatory factors [43]. Myc repression of C/EBPδ gene transcription may promote mammary tumorigenesis as C/EBPδ functions as a transcriptional activator of growth arrest, differentiation, apoptosis and inflammation related genes [3,44]. Myc repression is mediated by Myc interactions with promoter-bound transcriptional control proteins such as Sp1, Smads and Miz1 [25,44]. In this report, we provide new mechanistic insights into Myc repression of C/EBPδ gene expression. We demonstrate that Myc repression of the C/EBPδ promoter is dependent on Myc Box II (MBII), basic region (BR), helix-loop-helix (HLH) region and the leucine zipper (LZ) domains. In addition, we demonstrate that Myc repression of the C/EBPδ promoter is dependent on Miz1 and Max; two Myc interacting proteins that are constitutively associated with the C/EBPδ proximal promoter. Miz1 is required for Myc repression of C/EBPδ promoter activity but Miz1 does not activate the C/EBPδ promoter in nontransformed mammary epithelial cells. These results indicate that Miz1 functions exclusively in Myc mediated repression of C/EBPδ in nontransformed mammary epithelial cells. In addition, endogenous C/EBPδ expression is increased in cells treated with Miz1 and Max siRNAs, supporting a role for both Max and Miz1 in Myc repression of C/EBPδ expression. Finally, RuvBl1 (Pontin, TIP49) and RuvBl2 (Reptin, TIP48), two AAA+ family DNA helicases that interact with Myc Box II, repress C/EBPδ promoter activity [45]. These results provide new insights into Myc protein-protein interactions and the functional roles of Miz1, Max, RuvBl1 and RuvBl2 in Myc repression of C/EBPδ expression.

## Results

### Myc represses C/EBPδ promoter activity in nontransformed HC11 mammary epithelial cells

**Figure 1:**
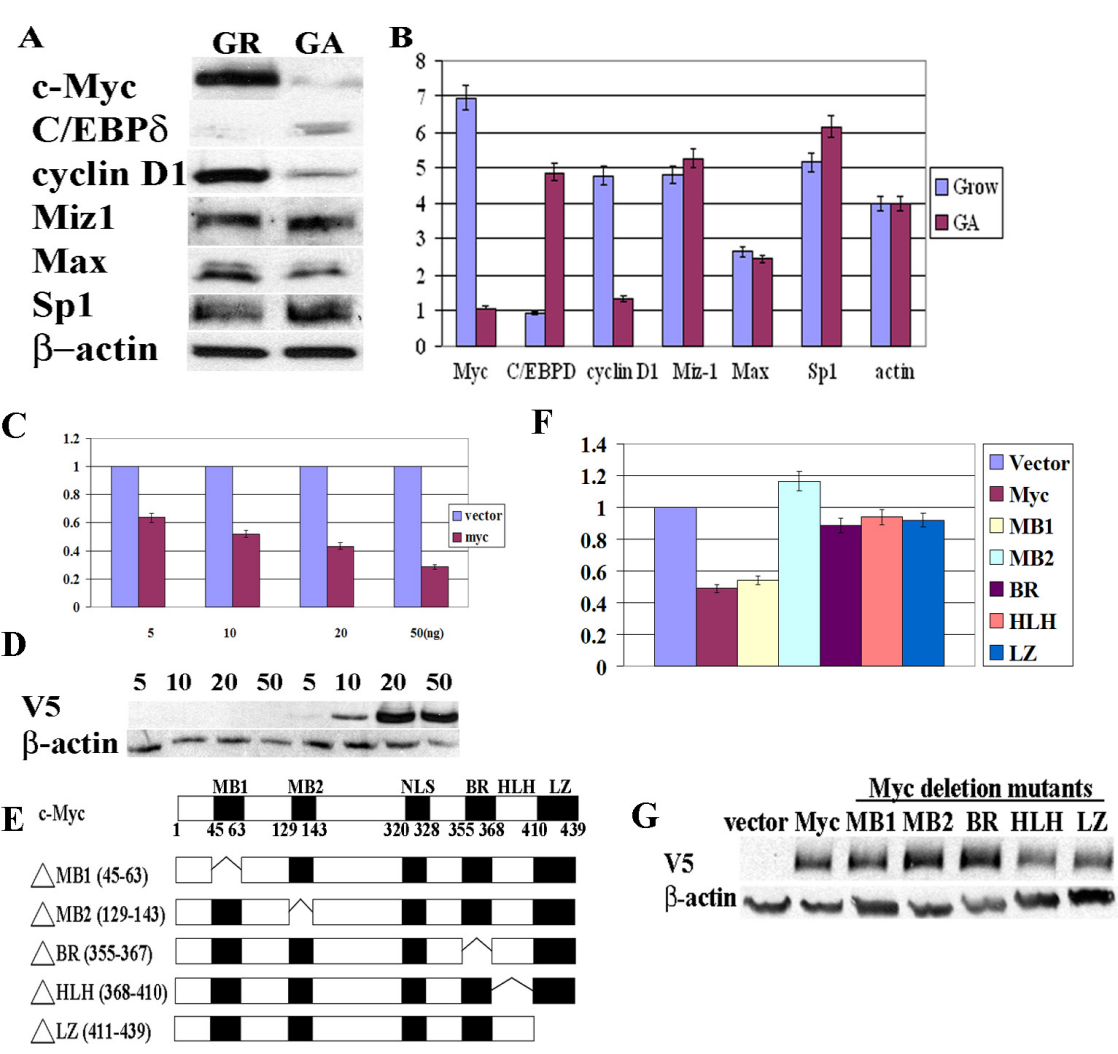
**Myc represses C/EBPδ promoter activation**. **A**. c-Myc (Myc), C/EBPδ, Cyclin D1, Miz1, Max and Sp1 protein levels in HC11 nontransformed mouse mammary epithelial cells under exponentially growing (GR) and growth arrest (GA) conditions (Western blot). **B**. Western blots from "A" were scanned to assess relative Myc, C/EBPδ, Cyclin D1, Miz1, Max and Sp1 protein levels in HC11 cells under Growing (Grow) vs Growth Arrest (GA) conditions. Due to differences in antibody affinity, quantitative comparisons are only valid for individual proteins under Grow vs GA conditions. **C**. HC11 cells were co-transfected with increasing amounts of a V5 tagged Myc expression construct (5-50 ng) plus a C/EBPδ promoter luciferase reporter construct. C/EBPδ promoter driven luciferase results were normalized to co-transfected Renilla luciferase control activity. Luciferase results from Myc treated cells are expressed relative to the vector control results, which were set as "1". **D**. Whole cell lysates (20 ul) from (**C**.) were immunoblotted and probed with an anti-V5 antibody to assess V5-tagged Myc protein levels in vector control (grey bar) and Myc transfected (black bar) HC11 cells. **E**. Schematic representation of Myc full length and Myc deletion mutants. Full length c-Myc contains: Myc box 1 (MB1, 45-63aa), MB2 (129-143aa), nuclear localization signal (NLS, 320-328aa), basic region (BR, 355-367aa), helix-loop-helix (HLH, 368-410aa) and leucine zipper (LZ, 411-439aa). **F**. HC11 cells were co-transfected with a C/EBPδ promoter luciferase reporter construct plus full length Myc or Myc deletion mutant expression constructs (V5 tagged). C/EBPδ promoter driven luciferase activities were normalized to co-transfected renilla luciferase control activity. C/EBPδ promoter driven luciferase results from Myc constructs are expressed relative to the vector control results, which were set as "1". **G**. Whole cell lysates from luciferase assays in (**D**.) were immunoblotted and probed with an anti-V5 antibody to assess Myc and Myc deletion mutant protein levels. All luciferase results shown are the average-fold changes relative to the vector control values from 2-3 independent experiments with duplicates performed in each experiment.

To investigate the role of Myc as a repressor of C/EBPδ gene transcription we first determined Myc and C/EBPδ protein levels in actively cycling (growing (GR)) and growth arrested (GA), nontransformed HC11 mammary epithelial cells. The results confirmed that Myc protein levels are elevated in growing HC11 cells and reduced in growth arrested HC11 cells (Figure 1AB). Conversely, C/EBPδ protein levels are virtually undetectable in growing HC11 cells and C/EBPδ protein levels are induced in growth arrested HC11 cells (Figure 1AB). Cyclin D1, a labile G1/S marker, is elevated in growing HC11 cells and reduced in growth arrested cells, paralleling Myc protein levels and confirming HC11 growth (cell cycle) status in these experiments [46,47]. Myc binding partners Miz1 and Max are also present at relatively constitutive levels in growing and growth arrested HC11 cells (Figure 1AB). Induction of C/EBPδ gene transcription requires the transcriptional activator Sp1 [7,48,49]. Sp1 protein levels, however, are unaffected by cell cycle status (Figure 1AB). These results demonstrate that Myc and C/EBPδ protein levels are directly influenced by growth status and that Myc and C/EBP protein levels are inversely correlated in nontransformed HC11 mammary epithelial cells.

To investigate Myc repression of C/EBPδ promoter activity, HC11 cells were transfected with increasing amounts of Myc (5~50 ng) plus a C/EBPδ promoter luciferase construct (Figure 1C). Myc repressed C/EBPδ promoter activity in a dose-dependent manner, even at dose levels as low as 5 ng (Figure 1C). Expression of Myc constructs was confirmed by Western blot analysis of cell lysates (Figure 1D). To map the domains of Myc essential for the repression of C/EBPδ promoter activity, Myc deletion mutants corresponding to Myc box 1 (ΔMB1, 45-63), Myc box 2 (ΔMB2, 129-143), basic region (ΔBR, 355-367), helix-loop-helix (ΔHLH, 368-410) and leucine zipper (ΔLZ, 411-439) were constructed (Figure 1E). The full length Myc construct and the Myc MB1 deletion mutant both repressed C/EBPδ promoter activity to ~50% of the empty vector control. These results indicate that the Myc MB1 deletion mutant is nearly as effective as the full length Myc construct in repressing C/EBPδ promoter activity and therefore, the Myc MB1 region is not required for Myc repression of C/EBPδ promoter activity (Figure 1F). In contrast, the Myc MB2, BR, HLH and LZ deletion mutants all resulted in C/EBPδ promoter activity that was similar to the empty vector control (Figure 1F). These results demonstrate that the MB2, BR, and the HLH/LZ regions are required for Myc repression of C/EBPδ promoter activity. Western blots demonstrated that the protein levels of the individual transfected Myc constructs were approximately equal; indicating that differences in C/EBPδ promoter activity were not due to variations in the expression of the transfected Myc constructs (Figure 1G).

### Miz1 (Myc-interacting zinc-finger protein1) is constitutively associated with the C/EBPδ promoter; Myc interacts with Miz1 in the repression of C/EBPδ promoter activity

**Figure 2:**
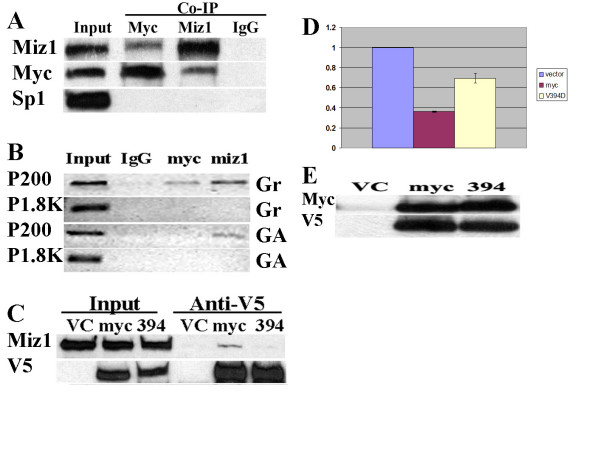
**Myc interacts with Miz1; Miz1 plays a key role in Myc repression of C/EBPδ promoter activity**. **A**. HC11 cell lysates were immunoprecipitated with anti-Myc and anti-Miz1 antibodies and the immunoprecipitates analyzed by Western blot using anti-Myc, Miz1 and Sp1 antibodies. "Input": western blot analysis of HC11 whole cell lysates (positive control). "IgG": nonspecific rabbit IgG immunoprecipitates (negative control). **B**. HC11 cell chromatin was immunoprecipitated using antibodies against Myc or Miz1. Immunoprecipitated DNA was amplified using primers flanking the C/EBPδ proximal (P200) promoter region and the distal (P1.8K) C/EBPδ upstream promoter regions. "Input" results are derived from direct PCR amplification of P200 and P1.8K C/EBPδ promoter regions from HC11 genomic DNA (Positive control). IgG: nonspecific rabbit IgG immunoprecipitated (negative control). **C**. HC11 cells were transfected with vector control, Myc wt or Myc V394D expression constructs (V5-tagged). Co-immunoprecipitation was performed using an anti-V5 antibody. Immunoprecipitates were analyzed by Western blot using anti-Miz1 and anti-V5 antibodies. **D**. HC11 cells were co-transfected with a C/EBPδ promoter luciferase reporter construct, vector control, Myc wild type (wt) or Myc V394D. C/EBPδ promoter driven luciferase activities were normalized to renilla luciferase activity. Results for the Myc transfected cells are expressed relative to the vector control results which were set as "1". **E**. Whole cell lysates (20 ul) from lucifease assays in (**D**.) were analyzed by Western blot to assess Myc wt and Myc V394D expression. Luciferase results shown are the average-fold changes relative to the vector control values from 2 independent experiments with duplicates performed in each experiment.

Myc represses gene promoters by interacting with DNA bound transcription factors including Sp1 and Miz1 [44]. To identify the Myc interacting protein implicated in Myc repression of the C/EBPδ promoter we transfected HC11 cells with V5-tagged Myc expression constructs and performed co-immunoprecipitations to assess Myc/Miz1 and Myc/Sp1 interactions in HC11 cell lysates. The results demonstrated that Myc interacts with Miz1, but not Sp1, supporting a role for Myc/Miz1 repression of the C/EBPδ promoter (Figure 2A). We next used ChIP assays to investigate the association between Myc and Miz1 and the C/EBPδ proximal promoter (P200) in Growing ("Gr", C/EBPδ non-expressing) and growth arrested ("GA", C/EBPδ expressing) HC11 cells. The ChIP results demonstrated that both Myc and Miz1 associate with the C/EBPδ proximal (P200) promoter in HC11 cells under Growing ("Gr", C/EBPδ non-expressing) conditions (Figure 2B). Miz1 remains associated with the C/EBPδ proximal (P200) promoter under growth arrest ("GA", C/EBPδ expressing) but Myc is not associated with the C/EBPδ proximal (P200) promoter in growth arrested (GA) HC11 cells (Figure 2B). Regardless of the growth conditions, neither Miz1 nor Myc is associated with the distal C/EBPδ promoter region located 1.8 kb upstream of the C/EBPδ transcription start site (P1.8K) (Figure 2B). These results are consistent with the presence a Myc/Miz1 complex in association with the C/EBPδ proximal promoter during active cell proliferation when C/EBPδ gene transcription is repressed and the absence of Myc in association with the C/EBPδ proximal promoter during growth arrest when C/EBPδ gene transcription is highly induced [6]. The negative ChIP results from the distal C/EBPδ promoter region 1.8 kb upstream of the C/EBPδ transcription start site (P1.8K) indicate that the Myc repressive complex is localized to C/EBPδ proximal (P200) region.

To determine if Myc/Miz1 interaction is required for Myc repression of C/EBPδ promoter activation we obtained a mutant Myc construct that is deficient in Miz1 binding (MycV394D, Val^394 ^→ Asp, generous gift from Dr. M. Eilers). To validate the MycV394D Miz1 binding defect co-immunoprecipitation assays were performed on HC11 cells transfected with V5 tagged Myc wild type (wt) or the MycV394D (Miz1 binding deficient) constructs. The results demonstrated that the Myc wt construct (myc) binds to Miz1, but the Myc V394D construct ("394") does not (Figure 2C). To assess the functional significance of Myc/Miz1 binding on Myc repression of C/EBPδ promoter activity HC11 cells were transfected with Myc wt or the MycV394D Miz1 binding deficient mutant plus a C/EBPδ proximal (P200) promoter-luciferase construct. The results demonstrated that the Miz1 binding deficient MycV394D mutant construct was relatively ineffective in repressing C/EBPδ promoter activity compared to the Myc wt construct (Figure 2D). Western blots documented the expression of transfected Myc constructs (Figure 2E). These results demonstrate that optimal Myc repression of C/EBPδ promoter activity requires Miz1.

### Miz1 does not activate the C/EBPδ promoter in nontransformed HC11 mammary epithelial cells

**Figure 3:**
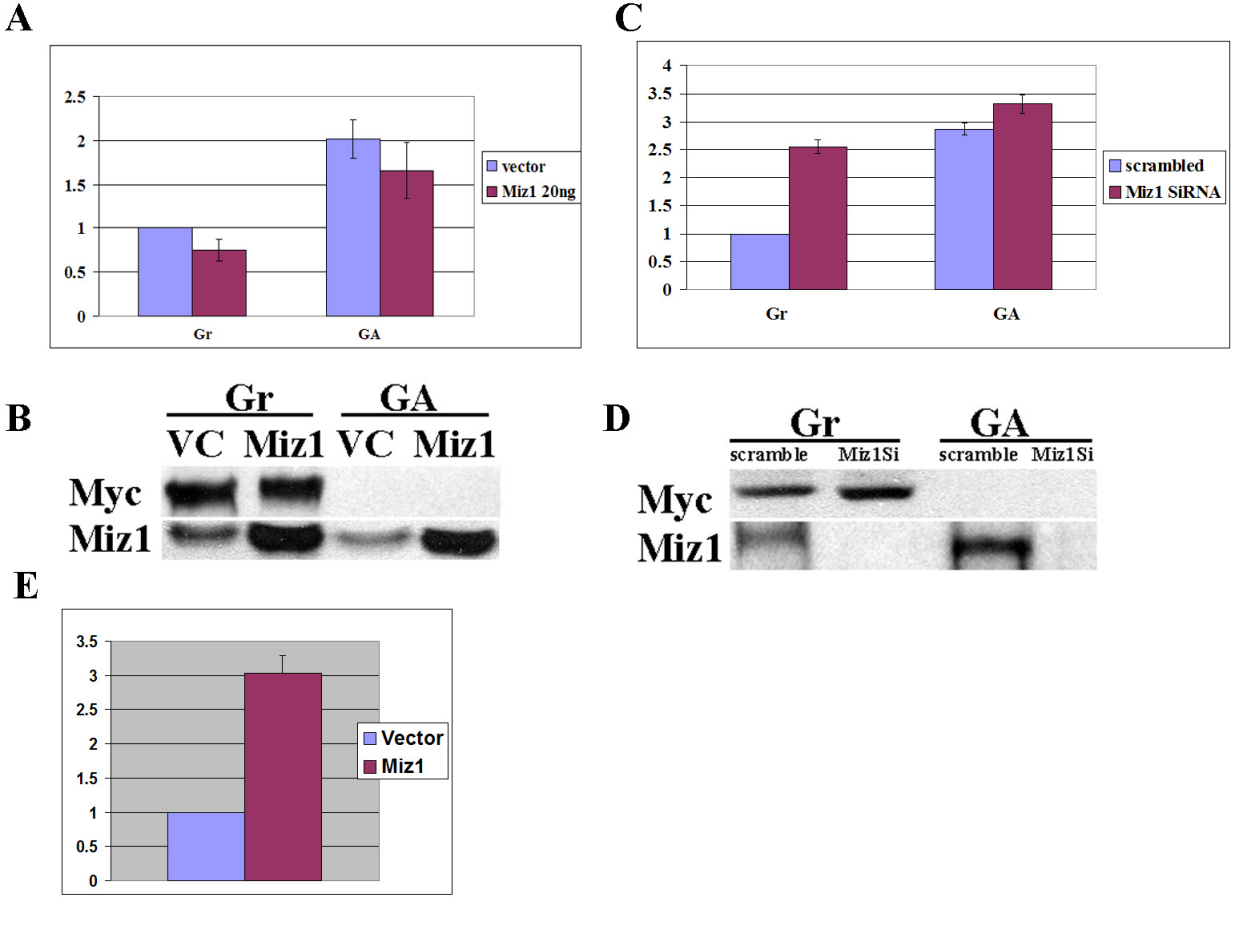
**Miz1 functions in Myc repression of C/EBPδ promoter activity**. **A**. HC11 cells were co-transfected with Miz1 or a vector control plus a C/EBPδ promoter luciferase construct and Renilla control. Luciferase activity was assessed under Growing (Gr) (Myc expressed, C/EBPδ repressed) or growth arrest (GA) (Myc not expressed, C/EBPδ expressing) conditions. Luciferase results were normalized to the Renilla control. C/EBPδ promoter driven luciferase results from Miz1 transfected cells are expressed relative to the vector control, "Gr" results which were set as "1". **B**. Lysates from luciferase assays (**A**.) were analyzed by Western blot to assess Myc and Miz1 protein levels. β-actin levels were assessed as a loading control. **C**. HC11 cells were transfected with Miz1 siRNA treatment and C/EBPδ promoter driven luciferase assays performed as described. Luciferase results were normalized to the Renilla control. C/EBPδ promoter driven luciferase results from Miz1 siRNA treated cells are expressed relative to the vector control, "Gr" results which were set as "1". **D**. Lysates from luciferase assays (**C**.) were analyzed by Western blot to assess Myc and Miz1 protein levels. β-actin levels were assessed as a loading control. Luciferase results for the Miz1 expression and Miz1 siRNA treatment groups shown are the average-fold changes relative to the "scrambled" siRNA values from 2 independent experiments with duplicates performed in each experiment.  **E.** HEPG2 cells were co-transfected with Miz1 or a vector control plus a low density lipoprotein receptor (LDLR) promoter luciferase construct and Renilla control. Luciferase results were normalized to the Renilla control. LDLR promoter driven luciferase results from Miz1 transfected cells are expressed relative to the vector control results which were set as "1".

Previous reports have demonstrated that Miz1 functions as a transcriptional activator and that Myc represses Miz1 target gene activation [39]. Miz1 is associated with the C/EBPδ proximal promoter during growth arrest (Figure 2B above) when C/EBPδ is actively transcribed [6], suggesting that Miz1 may function as a transcriptional activator of C/EBPδ transcription. To investigate Miz1 transcriptional activation of the C/EBPδ promoter HC11 cells were co-transfected with a Miz1 expression construct plus a C/EBPδ proximal promoter-luciferase (P200) construct. The results demonstrate that Miz1 expression does not increase C/EBPδ promoter activity in proliferating (growing, Gr), or in growth arrested (GA) HC11 cells (Figure 3A). As expected, C/EBPδ promoter activity is higher in growth-arrested vs growing HC11 cells [6,7]. Western blot analysis of HC11 cell lysates demonstrate the increased levels of Miz1 in HC11 cells transfected with the Miz1 expression construct and confirm the presence of Myc protein levels in growing (Gr) cells and the absence of Myc in growth arrested (GA) cells (Figure 3B).

Although Miz1 over expression had no effect on C/EBPδ promoter activity, reducing Miz1 levels by Miz1 siRNA treatment had a profound effect on C/EBPδ promoter activity in growing (Gr) HC11 cells. C/EBPδ promoter activity was induced ~2.5 fold in Miz1 siRNA treated; growing (Gr) HC11 cells (Figure 3C). Reducing Miz1 levels, however, had no effect on C/EBPδ promoter activity in growth arrested (GA) HC11 cells (Figure 3C). These results indicate that reducing Miz1 levels increases C/EBPδ promoter activity in proliferating cells, presumably by reducing Miz1/Myc repression (Figure 2D). Interestingly, C/EBPδ promoter activity is not altered by reducing Miz1 levels in growth arrested HC11 cells (Figure 3C). These results indicate that Miz1 does not increase C/EBPδ promoter activity during growth arrest. Western blot analysis of HC11 cell lysates confirmed that siRNA treatment was highly effective in reducing Miz1 protein levels (Figure 3D). To verify that Miz1 can function as a transcriptional activator in the proper cell context HEPG2 cells were transfected with a Miz1 expression construct plus a low-density lipoprotein receptor (LDLR) promoter-luciferase construct essentially as described by Tjian and co-workers [50]. The results demonstrated that Miz1 functions as a transcriptional activator of the LDLR promoter in HEPG2 cells (Figure 3E). These results demonstrate that Miz1 functions exclusively in Myc repression of C/EBPδ promoter activity under growing (proliferating) conditions but Miz1 does not activate C/EBPδ promoter activity in growth arrested HC11 nontransformed mammary epithelial cells.

### Miz1 binds to the -100 to -70 region of the C/EBPδ proximal promoter

**Figure 4:**
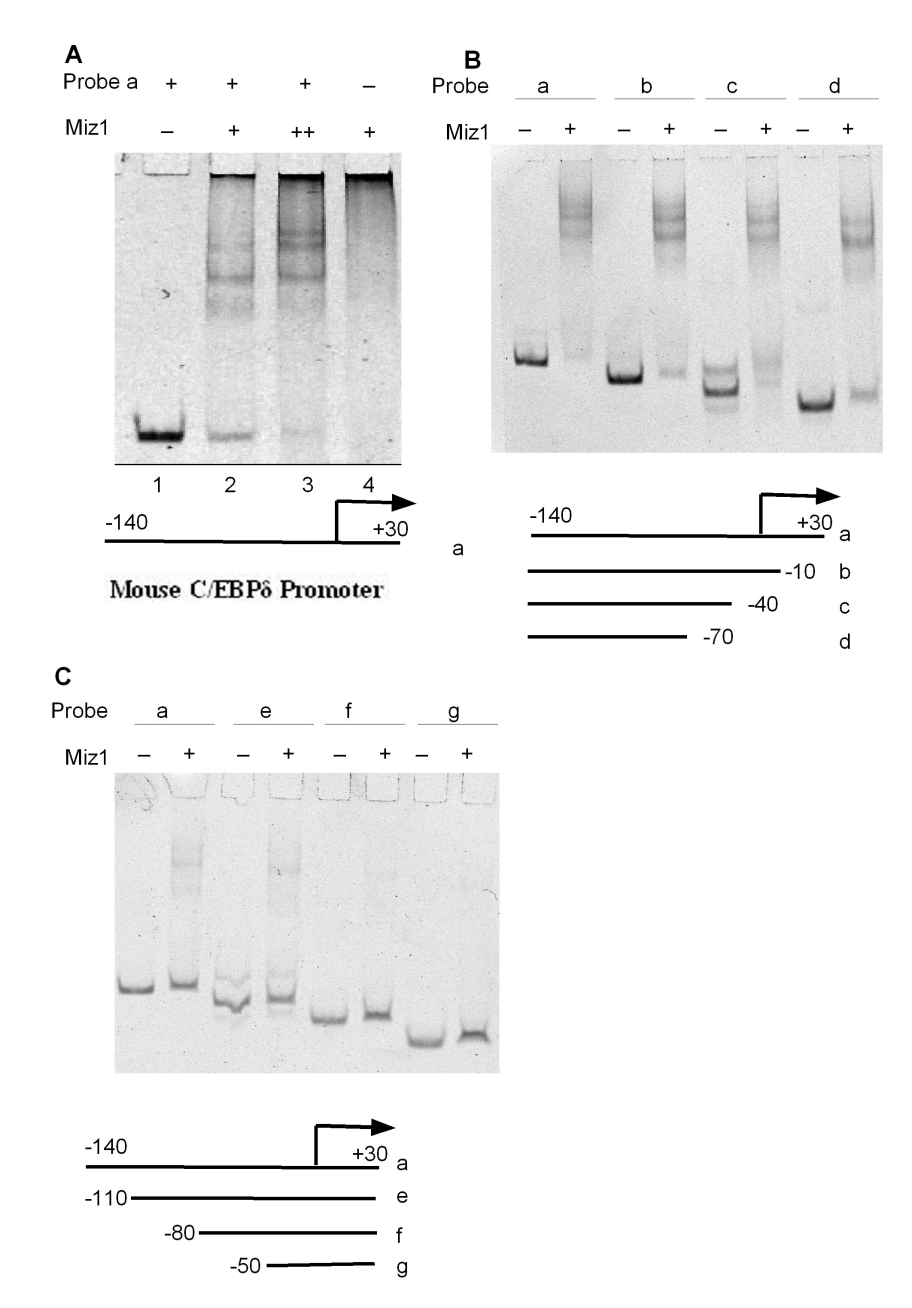
**Miz1 binds to the C/EBPδ proximal promoter**. **A**. Miz1 binds to the C/EBPδ promoter (170 bp, -140 to +30). Lanes: (1) C/EBPδ 170 bp promoter fragment; (2) Miz1 (5 ng) + C/EBPδ 170 bp promoter fragment; (3) Miz1 (15 ng) + C/EBPδ 170 bp promoter fragment; (4) Miz1 (5 ng). **B**. Miz1 binds to the C/EBPδ promoter 5' region. Lanes: a. C/EBPδ promoter fragment (-140 to +30), - or + Miz1 (5 ng); b. C/EBPδ promoter fragment (-140 to -10), - or + Miz1; c. C/EBPδ promoter fragment (-140 to -40), - or + Miz1; d. C/EBPδ promoter fragment (-140 to -70), - or + Miz1. **C**. Miz1 does not bind to C/EBPδ promoter fragments with the -110 to -80 region deleted. Lanes: a. C/EBPδ promoter fragment (-140 to +30), - or + Miz1 (5 ng); e. C/EBPδ promoter fragment (-110 to +30), - or + Miz1; f. C/EBPδ promoter fragment (-80 to +30), - or + Miz1; g. C/EBPδ promoter fragment (-50 to +30), - or + Miz1. Results are representative of 3 EMSA experiments.

**Figure 5:**
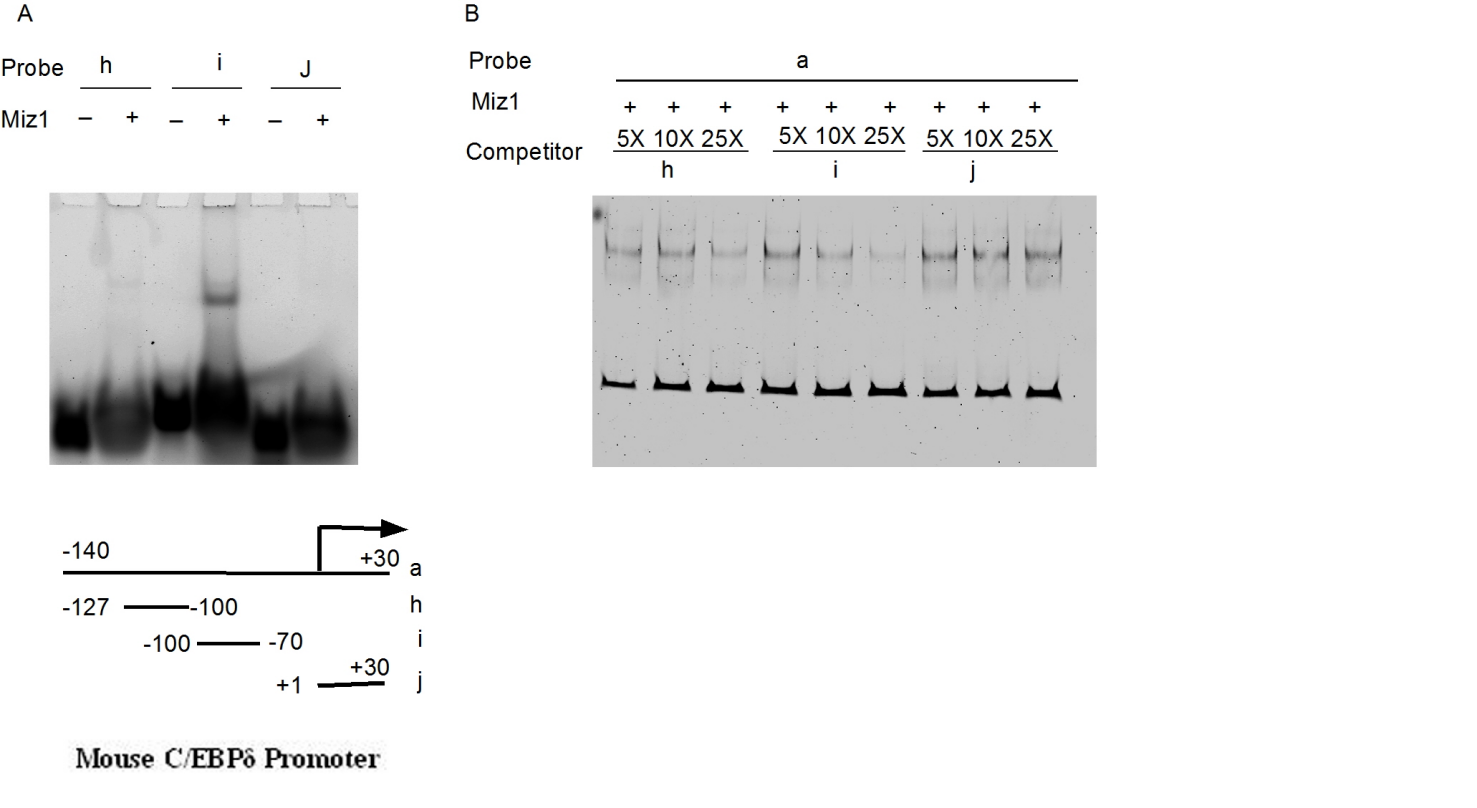
**Miz1 binding is localized to the -100 to -70 region of the C/EBPδ proximal promoter (Probe "i")**. **A**. EMSAs were performed without Miz1 (-) or with Miz1 (+) (5 ng) plus the following C/EBPδ proximal promoter fragments: -127 to -100 bp (Probe "h"); -100 to -70 (Probe "i"); or +1 to +30 (Probe "j") - or + Miz1 (5 ng). **B**. Competition EMSAs: C/EBPδ -100 to -70 proximal promoter fragment (Probe "i") effectively competes for Miz1 binding with the "full length" -140 to +30 C/EBPδ proximal promoter fragment. Miz1 was incubated with the "full length" -140 to +30 C/EBPδ proximal promoter fragment (Probe "a") plus 5×, 10× and 25× molar excess of C/EBPδ proximal promoter fragments: -127 to -100 bp (Probe "h"); -100 to -70 (Probe "i"); or +1 to +30 (Probe "j"). Results are representative of 2 EMSA experiments.

Myc functions as a transcriptional repressor by binding to DNA-bound Miz1 [28]. Miz1 binds to highly divergent proximal promoter transcription initiator (Inr) elements [28]. To investigate Miz1 binding to the C/EBPδ promoter we performed electromobility shift assays (EMSAs) using recombinant mouse Miz1 and fluorescent labeled C/EBPδ proximal promoter fragments. The initial results confirmed Miz1 binding to the -140 to +30 C/EBPδ proximal promoter (Figure 4A). To localize the region of Miz1 binding we performed EMSAs using C/EBPδ promoter fragments deleted from the 3' and 5' ends. Miz1 binding was retained in all C/EBPδ proximal promoter fragments deleted from the 3' end, indicating that Miz1 binding was localized within -140 to -70 region of the C/EBPδ proximal promoter (Figure 4B). Deletions from the 5' of the C/EBPδ proximal promoter indicated that the Miz1 binding was localized within the -110 to -80 region of the C/EBPδ proximal promoter (Figure 4C, lanes f, g). To further investigate Miz1 binding EMSAs were performed with recombinant Miz1 protein and short (~30 bp) C/EBPδ proximal promoter fragments spanning the following regions of the C/EBPδ proximal promoter: -127 to -100 bp (Probe "h"), -100 to -70 bp (Probe "i") and +1 to +30 (Probe "j", a negative EMSA control). The results demonstrated weak Miz1 binding to the -127 to -100 region ("h"), strong Miz1 binding to the -100 to -70 region ("i"), and no detectable Miz1 binding to the +1 to +30 region ("j") (Figure 5A). We next performed individual competition assays with the same 3 C/EBPδ promoter fragments and the C/EBPδ -140 to +30 proximal promoter fragment. The results demonstrated that the -100 to -70 ("i") C/EBPδ promoter fragment was the most effective in reducing Miz1 binding to the C/EBPδ -140 to +30 proximal promoter fragment (Figure 5B). The -127 to -100 C/EBPδ promoter exhibited a limited capacity to compete with the C/EBPδ -140 to +30 proximal promoter fragment for Miz1 binding, consistent with the weak binding to this region demonstrated in Figure 5A. Although Inr sequences are highly degenerate, a candidate Inr sequence is located at -85 to -93 (5'-CCCCAGTCCCT-3') of the C/EBPδ proximal promoter, within the -100 to -70 region [6].

### Max is constitutively associated with the C/EBPδ promoter and functions in the repression of C/EBPδ promoter activity

**Figure 6:**
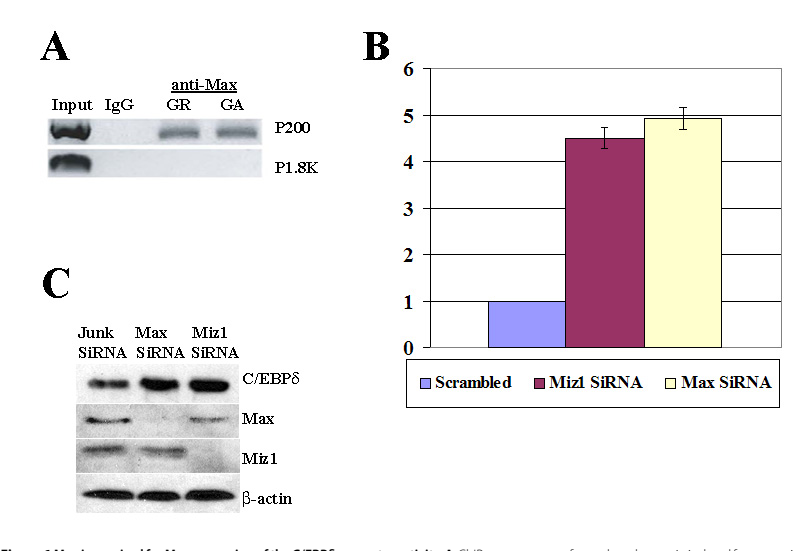
**Max is required for Myc repression of the C/EBPδ promoter activity**. **A**. ChIP assays were performed on chromatin isolated from growing (GR) and Growth arrest (GA) HC11 cells using anti-Max antibodies. Immunoprecipitated DNA was amplified using primers flanking the C/EBPδ proximal promoter region (P200) and the C/EBPδ upstream promoter region (P1.8K). "Input" results are derived from PCR amplification of HC11 genomic DNA. Normal rabbit IgG was used as negative control. **B**. Growing HC11 cells were nucleofected with the "scrambled" siRNA control, Miz1 siRNA and Max siRNA. C/EBPδ promoter-luciferase results were normalized to the Renilla control. The luciferase results for the "scrambled" siRNA control were set as "1". Luciferase results for the Miz1 and Max siRNA treatment groups shown are the average-fold changes relative to the "scrambled" siRNA control values from 3 independent experiments with duplicates performed in each experiment (n = 6). **C**. HC11 cells were nucleofected with a scrambled siRNA, Max or Miz1 siRNA constructs using the Amaxa nucleofector protocol. Nucleofected HC11 cells were then cultured in complete growth media (proliferating, growing conditions) in the presence of OSM (GROW + OSM). Whole cell lysates were isolated and analyzed by Western blot using anti- C/EBPδ, Max and Miz1 antibodies (lane 1-3). β-actin was assessed as the loading control. The results shown are representative of three independent experiments.

Myc Associated protein X (Max) is a ubiquitously expressed, long lived (t 1/2 > 24 hours) helix loop helix/leucine zipper (HLH/bZIP) protein that heterodimerizes with Myc and is required for Myc transcriptional activation and repression [51-53]. Using ChIP assays, we assessed the association between Max and the C/EBPδ promoter under growing and under growth arrest conditions. The results indicated that Max is associated with the C/EBPδ promoter under both growing (GR, C/EBPδ expression repressed) and growth arrest (GA, C/EBPδ expression highly induced) conditions (Figure 6A). To determine if Max is required for Myc repression of C/EBPδ promoter activity HC11 cells were treated with Max siRNA and C/EBPδ promoter driven luciferase activity assessed. The results indicated that Max siRNA treatment reduces Myc repression of C/EBPδ promoter activity and this reduction in Myc repression is comparable to Miz1 siRNA treatment (Figure 6B). These results demonstrate that Max is constitutively associated with the C/EBPδ promoter and plays a key role in Myc repression of C/EBPδ promoter activity.

IL-6 and Oncostatin M (OSM) induce STAT3 activation (phosphorylation) and phosphorylated STAT3 (pSTAT3) activates C/EBPδ transcription in growth arrested cells [7,8,54]. OSM activates pSTAT3, but pSTAT3 does not fully activate C/EBPδ expression in proliferating (growing) cells due to Myc repression [43]. Myc repression of OSM induced endogenous C/EBPδ expression is attenuated by Myc siRNA treatment [43]. To investigate the role of Max and Miz1 in Myc repression of endogenous C/EBPδ expression HC11 cells were transfected with Max and Miz1-specific siRNAs. Endogenous C/EBPδ expression was assessed by western blot of whole cell lysates from actively proliferating (growing) vector control, Max and Miz1 siRNA treated HC11 cells treated with OSM. The results demonstrated that Max and Miz1 siRNA treatments reduced endogenous Max and Miz1 protein levels and the individual reductions in Max and Miz1 protein levels were associated with increased OSM-induced C/EBPδ protein levels compared to scrambled or "Junk" siRNA treated HC11 cells (Figure 6C). These results indicate that Max and Miz1 function in repression of endogenous C/EBPδ gene expression.

### RuvBl1(Pontin, TIP49) and RuvBl2 (Reptin, TIP48) repress C/EBPδ promoter activity

**Figure 7:**
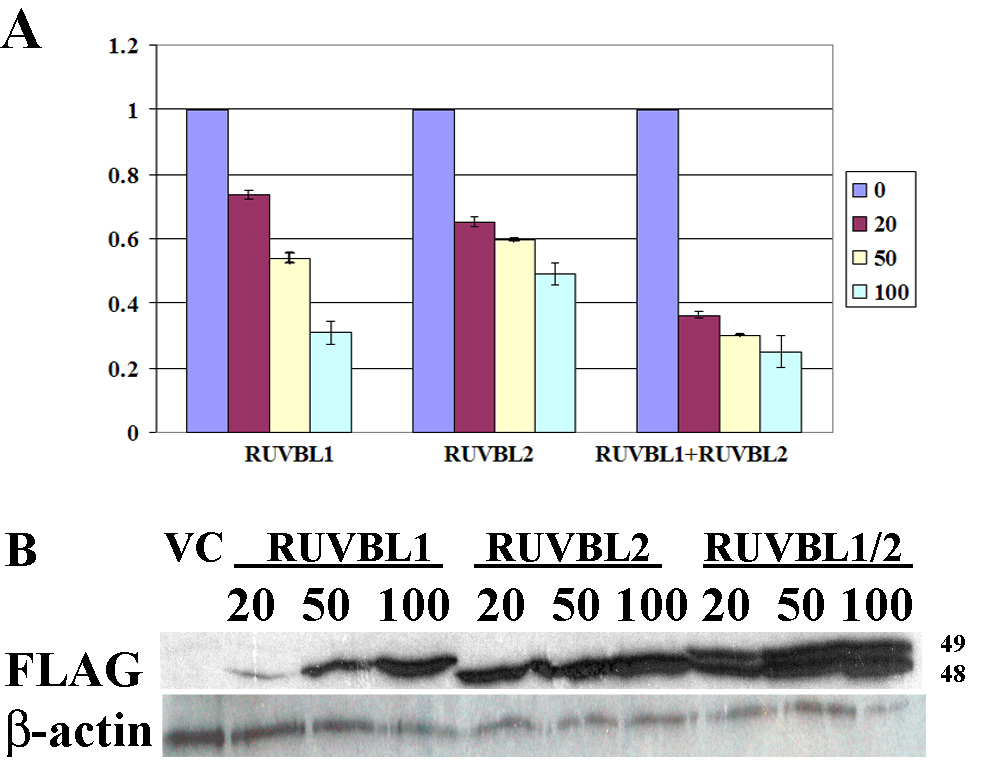
**RuvBl1 and RuvBl2 repress C/EBPδ promoter activity**. **A**. Exponentially growing HC11 cells were co-transfected with vector control (VC) or increasing amounts (20, 50 100 μg) of FLAG-tagged RuvBl1 (TIP49, Pontin), RuvBl2 (TIP48, Reptin) or RuvBl1/RuvBl2 combined plus the C/EBPδ proximal promoter-luciferase and the Renilla control. Luciferase results were normalized to the Renilla control, the VC values were set as "1" and the RuvBl1 and RuvBl2 transfected cell luciferase results are expressed relative to the VC control. **B**. Lysates from luciferase assays (**A**.) were analyzed by Western blot to using an anti-FLAG antibody to assess Myc and Miz1 protein levels. β-actin levels were assessed as a loading control. All luciferase results shown are from 3 independent experiments with duplicates performed in each experiment.

RuvBl1 (Pontin, TIP49) and RuvBl2 (Reptin, TIP48) are members of the highly conserved AAA+ (ATPases associated with diverse cellular activities) superfamily with functions in chromatin remodeling and transcriptional regulation [45]. We hypothesized that RuvBl1 (Pontin, TIP49) and RuvBl2 (Reptin, TIP48) may contribute to Myc repression of C/EBPδ promoter activity as both proteins interact with Myc Box II and enhance Myc transcriptional repression and Myc mediated transformation [25,34,53,55]. In addition, RuvBl1 (Pontin) and RuvBl2 (Reptin) are overexpressed in a variety of human cancers [45]. To investigate the role of RuvBl1 and RuvBl2 in Myc repression of C/EBPδ promoter activation RuvBl1 and RuvBl2 expression constructs were transfected into proliferating HC11 cells and C/EBPδ promoter activity assessed by luciferase assay. The results demonstrated that both RuvBl1 and RuvBl2 repress C/EBPδ promoter activity in a dose-dependent manner (Figure 7A). In addition to assessing the repressive effects of RuvBl1 and RuvBl2 individually, the repressive effect of co-transfecting RuvBl1 and RuvBl2 on C/EBPδ promoter activity was also investigated. The results demonstrated that co-expression of RuvBl1 and RuvBl2 was more effective in repressing C/EBPδ promoter activity than expression of either RuvBl1 and RuvBl2 alone (Figure 7A). Western blot analysis documented the expression of the transfected constructs and the positive correlation between increased RuvBl1 and RuvBl2 expression levels and increased repression of C/EBPδ promoter activity (Figure 7B).

## Discussion

The findings from this study demonstrate that Myc represses C/EBPδ expression by associating with the C/EBPδ proximal promoter as transient component of a multi-protein repressive complex. Transcriptional repression is a major mechanism of Myc oncogenesis and Myc repressed genes include critical regulators of cell cycle progression, growth arrest and differentiation such as p21^*CIP*1^, p27^*KIP*1^, p15^*INK*4^, p18^*INK*4*c*^, p57^*KIP*2^, gas1, and C/EBPα [44]. Myc repression of C/EBPδ transcription is Miz1 dependent, indicating that Myc repression of C/EBPδ transcription parallels Myc repression of p15^*INK*^, p21^*CIP*1^, p27^*KIP*1^, Mad4 and C/EBPα [25]. However, Miz1 does not function as a transcriptional activator of the C/EBPδ promoter in nontransformed mammary epithelial cells, differentiating Miz1 function in the regulation of C/EBPδ from p15^*INK*^, p21^*CIP*1 ^and Mad4 [44]. Although Miz1 does not activate the C/EBPδ promoter, ChIP assays demonstrated that Miz1 is constitutively associated with the C/EBPδ promoter. EMSA analysis localized the Miz1 binding site to the -100 to -70 region of the C/EBPδ proximal promoter. This a region contains a candidate Inr (-85 to -93) immediately downstream of STAT3/Sp1 consensus sites (-120 to -104) that are associated with C/EBPδ transcriptional activation [7,54]. We and others have reported that pSTAT3 is a potent transcriptional activator of C/EBPδ gene expression [7,8]. The presence of the Miz1 binding site downstream of the C/EBPδ consensus transcriptional activation sites provides a rationale for how Myc represses C/EBPδ expression in actively cycling cells that exhibit increased pSTAT3 in response to IL-6 family cytokines [43]. These findings suggest that Myc repression of C/EBPδ expression could contribute to the cascade of Myc mediated events that result in aberrant cell proliferation and enhanced transformation.

Max, a well-established Myc binding partner, also plays a key role in Myc repression of C/EBPδ expression. Like Miz1, Max is constitutively associated with the C/EBPδ promoter even in the absence of Myc, a finding that is consistent with a previous report by Mao, et al, [52]. The recruitment of Miz1 and Max to the C/EBPδ proximal promoter may be facilitated by the C/EBPδ proximal promoter "open" chromatin conformation [43]. We previously reported that the C/EBPδ proximal promoter is in an open chromatin conformation and "pre-loaded" with transcriptional machinery components associated with transcriptional activation including Sp1, cyclic AMP response element-binding protein (CREB), TATAA Binding protein (TBP) and RNA Pol II [43]. The present results demonstrate that Miz1 and Max, two proteins that function in C/EBPδ transcriptional repression, are also constitutively associated with the C/EBPδ promoter. These results are consistent with a model in which the C/EBPδ proximal promoter exists in a unique state, poised for activation or repression by the constitutive presence of proteins that mediate both transcriptional activation and repression.

Although Myc transcriptional repression is critical for Myc mediated cell transformation, the proteins that interact with Myc and function in gene repression are poorly characterized. RuvBl1 (Pontin, TIP49) and RuvBl2 (Reptin, TIP48) are two AAA+ ATPase helicases that interact with Myc Box II and function in Myc transcriptional repression, and have been shown to increase cell proliferation and transformation [55-57]. Individually, both RuvBl1 and RuvBl2 repressed C/EBPδ promoter activity, however, co-expression of RuvBl1 and RuvBl2 was most effective in repressing C/EBPδ promoter activity. This suggests that Myc transcriptional repression of C/EBPδ may be mediated by a multi-protein complex composed of DNA bound Miz1, Myc/Max and possibly RuvBl1 and RuvBl2. Studies in *Xenopus *demonstrated that RUVBL1/RUVBL2 (xPontin/xReptin) induce cell proliferation during embryogenesis by enhancing Myc repression of p21 [58]. Our findings suggest that a similar mechanism may mediate Myc repression of C/EBPδ and possibly other growth suppressor genes (such as p21^*Waf*1/*CIP*1^), in promoting aberrant mammary epithelial cell proliferation and transformation.

Despite the critical role of Myc transcriptional repression in cell transformation, the mechanism by which Myc transcriptional repression leads to cell transformation is poorly understood. Several lines of evidence indicate that Myc can recruit DNA methyltransferases and that Myc transcriptional repression can progress to transcriptional silencing. For example, Myc repression of p21^*Waf*1/*CIP1 *^transcription in human U2OS osteosarcoma cells occurs via formation of a repressive complex including Myc, Miz1 and DNA Methyltransferase3a (Dnmt3a) [59]. In addition, studies in human cervical and hepatocellular carcinoma cells have shown recruitment of DNA methyltransferases and silencing of the human C/EBPδ (CEBPD) promoter by hypermethylation [21]. Our lab reported that the C/EBPδ gene is silenced by promoter hypermethylation in the SUM-52PE human breast cancer cell line and that primary breast tumors exhibiting reduced C/EBPδ expression are characterized by site-specific promoter methylation [10,11,54]. The results from this study demonstrate that Myc repression of C/EBPδ transcription is a regulated process that is coordinated with cell cycle status in nontransformed cells. Further studies are needed to determine how this regulated Myc repression function is altered and progresses to gene silencing and cell transformation.

## Conclusion

The results of this study identify protein-protein and DNA-protein interactions that mediate Myc repression of C/EBPδ gene expression. These results extend current working models of Myc transcriptional repression and suggest future directions to pursue in the characterization of the network of proteins that function in Myc transcriptional repression. The results presented have focused on Myc repression of the mouse C/EBPδ promoter in HC11 mouse nontransformed mammary epithelial cells; however, human Myc expression constructs also repress the human C/EBPδ promoter in nontransformed human mammary epithelial cells (MCF-10A) (data not shown). Current experiments are focused on further characterizing Myc interacting proteins, deciphering the sequence of events that mediate Myc repression of C/EBPδ in nontransformed mouse and human cells, and determining how this sequence progresses to gene silencing and cell transformation. Defining the protein interactions that mediate Myc repression, and the role of Myc in the silencing of tumor suppressor genes, will facilitate the development of pharmacological interventions to inhibit the functions of Myc that promote cell transformation.

## Methods

### Cell Culture

HC11 mouse mammary epithelial cells were grown in complete growth media (CGM) containing RPMI 1640 medium plus 5% fetal bovine serum (FBS), 10 μg/ml bovine insulin, 10 ng/ml epidermal growth factor, 100 U/ml penicillin, 100 μg/ml streptomycin and 500 ng/ml Fungizone. Growth arrest was induced by 24~48 hrs serum and growth factor withdrawal (growth arrest medium, GAM, 0.1% FBS).

### Plasmid Constructs

Mouse C/EBPδ proximal promoter sequence flanking -127 bp to transcriptional start site (P-127, containing Sp1, STAT3 and CREB binding sites) was constructed in the pGL2 basic luciferase reporter vector [7,60]. Myc and MycV394D mutant constructs in pBabe-puro vector were a generous gift from Dr. Martin Eilers (Institute for Molecular Biology and Tumor Research, University of Marburg, Germany). Myc and MycV394D were then amplified by PCR from pBabe-puro vector using primers specific for Myc. The primer sequences for Myc wild type and MycV394D cloning are as follows: 5'-CGCGGATCCGCGATGCCCCTCAACGTTAGCTTC-3' (forward primer) and 5'-GCTCTAGACGCGCACAAGAGTTCCGTAGCTG-3' (reverse primer). Myc deletion constructs MycΔ45-63(MB1), MycΔ129-143(MB2), MycΔ355-367(BR), MycΔ368-410(HLH) and MycΔ411-439(LZ) were constructed by site-specific mutagenesis as previously described [61,62]. Myc-, Myc deletion- and V394D- pcDNA3.1-V5-His expression constructs were verified by sequencing. The Miz1 full length cDNA construct in pCMV6 vector was purchased from Origene.

### Transfection Protocol

HC11 cells were plated in 12-well plates, grown to 50% confluence in CGM and transfected using the enhanced Lipofectamine transfection protocol (Invitrogen, Carlsbad, CA) as previously described [60]. Co-transfections were performed with 0.3 ug C/EBPδ promoter luciferase reporter construct, 1 ng Renilla luciferase reporter construct (transfection efficiency control), and 5~50 ng of expression constructs or vector controls. For growth arrest experiments, transfected cells were washed 2× with PBS and cultured in GAM for 24-48 hours. Cells were harvested and assayed for firefly and renilla luciferase activities using the Dual-Luciferase Reporter Assay kit with luciferase detection by Hewlett-Packard Lumicount microplate luminometer as previously described [43]. C/EBPδ promoter activities were normalized to renilla luciferase activity. Results shown are the average-fold changes from 3 independent experiments with duplicates. Co-immunoprecipitation experiments were performed as described [43,61,62]. HC11 cell lysates used in co-immunoprecipitation assays were prepared by transfecting Myc or V394D Myc mutant expression constructs (1 μg) (Lipofectamine) into HC11 cells. HC11 Miz1 and Max siRNA transfections were performed using the Amaxa Nucleofector (Amaxa, Inc., Cologne, Germany). Briefly, HC11 cells were suspended in Amaxa Nucleofector Solution V supplemented with 50 pmol Miz1 or Max Smartpool siRNAs (Dharmacon, Inc., Lafayette, CO) and the nucleofection was performed using cell-type specific protocol (T-20). HC11 cells nucleofected with non-specific scrambled siRNAs were used as controls. Transient siRNA nucleofection protocols were optimized and protocols achieving >80% specific gene knockdown as verified by western blot were used in all experiments.

### Western blot and co-immunoprecipitation assays

Western blots were performed on whole cell lysates as previously described [61,62]. Co-immunoprecipitation assays were performed with HC11 cell lysates isolated by NP-40 lysis, primary antibody immunoprecipitation, Protein A-Agarose bead pull down, elution and analysis by SDS PAGE as previously described [61]. Co-immunoprecipitations were performed 2-3 times and representative results presented.

### Chromatin immunoprecipitation (ChIP)

ChIP experiments were performed using the Chromatin Immunoprecipitation (ChIP) Assay Kit (Sigma) as previously described [3,43]. Briefly, HC11 cells were cross-linked with 1% formaldehyde, washed 3× with cold PBS (4°C), and the nuclear pellets were collected by centrifuge. Nuclear pellets were then resuspended in 300 μl DNA shearing buffer containing protease inhibitor cocktail, sonicated on ice to approximately 200~1000 bp (verified by standard agarose gel analysis), centrifuged at 14000 rpm for 10 minutes to pellet cell debris and the supernatants were collected and diluted 1:1 in dilution buffer and used for DNA immunoprecipitation. 10 ul diluted supernatant was used as input control. One μg of Myc or Miz1 specific IgG immunoprecipitated protein-DNA complexes were isolated and protein-DNA crosslinks reversed (65°C, 2 hours). After purification, immunoprecipitated DNA was analyzed by PCR using primers specific for proximal and distal mouse C/EBPδ promoter [60]. Primer sequences are as follows: P200 (region -226 to -24 of the mouse C/EBPδ promoter containing STAT3 and SP1 binding sites), 5'-GCGTGTCGGGGCCAAATCCA-3'(forward primer), 5'-TTTCTAGCCCCAGCTGACGCGC-3'(reverse primer); P1.8K (region -1856 to -1676 of the promoter) as control, 5'-TGCTTCTATGGCATCCAG-3'(forward primer), 5'-GAGGGGCTGTGGAATATT-3'(reverse primer).

### Miz1 protein purification

Full length Miz1 cDNA was cloned into pGEX-4T-1 vector (Miz1-GST). The Miz1-GST plasmid was transformed to BL21 (DE3) competent cell (Stratagene). The Miz1-GST protein was purified by affinity binding using Glutathione sepharose beads (GE Healthcare) following the manufacturer's protocol. Miz1 protein was confirmed by western blot with detection using Miz1 and GST antibodies (Santa Cruz, Biotechnology).

### Electromobility Shift Assay (EMSA)

DNA probes (a to g) were generated by PCR using mouse C/EBPδ promoter (1.7 kb fragment) as template. Primer sequences are available upon request. Double stranded oligos used to produce Probes h, j, and i were purchased (Sigma). Probes used in EMSA reactions were 5' end-labeled with 6-FAM (6-Carboxyfluorescein, Sigma). EMSAs were performed by incubating labeled probes (20 ng) with purified Miz1 protein in binding buffer (10 mM Tris pH 7.9, 4 mM MgCl_2, _5% glycerol, 0.1 mM DTT, 20 ng/μl poly(dI:dC) and 0.2% NP-40) for one hour at room temperature. To perform EMSA competition assays unlabelled probes were pre-incubated with Miz1 in binding buffer for 10 min prior to addition of the labeled probe. The concentration of unlabeled probes used was 5-25-fold molar excess over labeled probe. Following incubation, samples were loaded onto a 4.5% native acrylamide gel (pre-run for one hour) and electrophoresed for one hour at 100 V. Gels were scanned using the Typhoon 9410 imager (GE healthcare).

## List of abbreviations

C/EBPδ: CCAAT/Enhancer Binding Proteinδ; ChIP: Chromatin Immunoprecipitation; MECs: Mammary epithelial cells; SAGE: Serial Analysis of Gene Expression; Inr: initiator element; EMSA: electromobility shift assay; Miz1: Miz1 (Myc-interacting zinc-finger protein 1); Max: Myc Associated protein X; CREB: cyclic AMP response element-binding; Dnmt3a: DNA Methyltransferase3a.

## Competing interests

The authors declare that they have no competing interests.

## Authors' contributions

All authors contributed to the experimental design, data interpretation, and manuscript development. JS, XY and YZ carried out the experiments, initial data analysis and figure design and optimization. JD advised on all experimental design aspects, data interpretation and final manuscript form. All authors have read and approve of the contents of this manuscript.
